# D.A.R.E./keepin’ it REAL elementary curriculum: Substance use outcomes

**DOI:** 10.1371/journal.pone.0284457

**Published:** 2023-04-28

**Authors:** William B. Hansen, Emily R. Beamon, Santiago Saldana, Samantha Kelly, David L. Wyrick

**Affiliations:** 1 Prevention Strategies, Greensboro, North Carolina, United States of America; 2 Department of Public Health Education, University of North Carolina at Greensboro, Greensboro, North Carolina, United States of America; 3 Department of Biostatistics and Data Science, Wake Forest University School of Medicine, Winston-Salem, North Carolina, United States of America; University of Pennsylvania, UNITED STATES

## Abstract

In response to a need to implement an evidence-based prevention program, D.A.R.E. America adopted *keepin’ it REAL*. The program was previously developed and tested in middle school settings. As part of its adoption, an elementary version of the program was developed. This study tests the effectiveness of *keepin’ it REAL* when delivered to fifth graders. The intervention was delivered to two cohorts of students, the first in the 2019–2020 school year, the second in the 2020–2021 school year. Pretest surveys were completed by 6,122 students. The COVID-19 pandemic interfered with posttest and follow-up data collection. At immediate posttest, 2,049 students (33.5%) completed analyzable posttest surveys. One year after the pretest, 1,486 (24.3%) students completed usable follow-up surveys. We used algorithmically generated cases (virtual controls) that use treatment cases’ pretest psychosocial scores to assess program effectiveness. When compared to virtual control cases, the program had identifiable improvements in both a key psychosocial measure and in terms of deterring the onset of 30-day alcohol use, drunkenness, and vaping. Outcomes suggest that the delivery of elementary school *keepin’ it REAL* by D.A.R.E. officers is having a positive effect in terms of deterring the onset of alcohol use and vaping.

## Introduction

D.A.R.E. America has delivered drug prevention programming to America’s youth since 1983. Evaluations of the first generation D.A.R.E. program began in the 1980s [1] and continued through the 1990s [2–8]. Eventually, there were sufficient numbers of studies that multiple meta-analyses were completed [9–12]. The general consensus of research on the first generation D.A.R.E. curriculum was that the program resulted in either neutral or small, positive effects.

While independently conducted evaluations have provided mixed findings, D.A.R.E. has been incredibly successful in building a community-based infrastructure. This has included long-standing collaborations between local k-12 schools and law enforcement for both providing drug prevention training for officers and delivering school-based prevention programming across the country [13]. D.A.R.E. program have been widely adopted. More than 6,000 law enforcement agencies currently deliver D.A.R.E. programs to more than 1.2 million K-12 students who reside in more than 10,000 communities throughout the United States. It is estimated that there are 29 international D.A.R.E. programs that reach an additional 3 million students annually. The breadth and longevity of adoption underscores the need to continue evaluating D.A.R.E.’s effectiveness.

In 2012, the D.A.R.E. Scientific Advisory Board advised D.A.R.E. America to adopt *keepin’ it REAL* (*kiR*), a drug and alcohol prevention program certified as evidence-based program to prevent adolescent drug use [14–18] and recommended by the U.S. Surgeon General [19] as an evidence-based program to prevent adolescent drug use. Staff from D.A.R.E. America (including members of the Scientific Advisory Board) worked collaboratively with *kiR* developers, Drs. Hecht and Miller-Day, to adapt the curriculum to make it appropriate for delivery by D.A.R.E. certified officers to 5^th^ grade elementary school students. In its current form, local law enforcement officers must pass a rigorous 80-hour training to become a D.A.R.E. certified officer. The adapted program delivered by D.A.R.E. officers has shown promise in terms of improving targeted mediators [20, 21]. While the adoption of *kiR* has provided D.A.R.E. with an evidence-based program [22], Caputi and McLellan [23, 24] have argued that there is not yet sufficient evidence that D.A.R.E. officers’ ability to deliver the program has been demonstrated. This argument has been countered by Miller-Day and Hecht [25].

The elementary *kiR* program consists of 10 45-minute lessons that also include take-home family talk activities. The curriculum identifies fundamental, basic skills and developmental processes needed for healthy development including: (1) self-awareness and management, (2) responsible decision making, (3) understanding others, (4) relationship and communication skills, and (5) handling responsibilities and challenges.

Compared to the original D.A.R.E. program, which relied heavily on didactic elements, the *kiR* version is designed to be more interactive. Based on published research [26], this relies on systematically developing a classroom environment that provides emotional support. In practice *kiR* includes more questions designed to engage in discussion, demonstration, and role play [27].

### The current study

In 2018, D.A.R.E. America entered into a contract with UNC Greensboro (UNCG) and Prevention Strategies to complete an independent evaluation of D.A.R.E. officers’ delivery of *kiR* in elementary schools. The goal of the current study is to document behavioral outcomes achieved through the implementation of the D.A.R.E. *kiR* elementary school program as delivered by certified D.A.R.E. officers in the classroom.

## Methods

### Human subjects protection

The University of North Carolina at Greensboro Institutional Review Board approved the project’s plan for ensuring students’ voluntary participation and safety. The IRB waived written and verbal informed consent. Parents were informed about the study via written notification that included an opportunity to review student surveys and given the option to exclude their student from participation in the surveys (passive informed consent). Students did not complete written or verbal consent but were given the opportunity to opt out of participating in surveys or to withdraw from participating at any time (passive assent). Because school personnel administered surveys, schools were responsible for overseeing parental and student withdrawal from the study. Documentation of exclusion and withdrawal to the research team was not required by the IRB. Students were identified by an ID number that was linked to their names but names were known only to their classroom teachers.

### Participants

The evaluation included students, classrooms, and schools and included participants from two grade cohorts. In Year 1 (2019–2020), we recruited 3,490 5^th^ graders from 40 elementary schools and 151 classrooms. In Year 2 (2020–2021), 2,632 students participated. These students were from 37 schools (the same schools from Year 1) and 141 classrooms. In all, 6,122 students across 292 classrooms completed pretest surveys. Schools were located in Arizona, Georgia, Iowa, New Mexico, New York, North Carolina, Ohio, Tennessee, and Utah.

The average age of students at pretest was 10.82 years. Genders were roughly equally represented: 48.5% males and 51.5% females. Most were not Hispanic (85.1%), were White (59.4%), with smaller numbers of Black/African American (17.3%), and the remainder being Asian, Pacific Islander, Native American, claiming multiple races, or other (23.3%).

A total of 23 D.A.R.E. officers taught the program, 12 participated in both years, 6 participated only in Year 1 and 5 participated only in Year 2. Officers ranged in age from 31 to 65 years, with an average age of 43.

### Implementation during the pandemic

Year 1 started in the fall of 2019. COVID occurred during the spring semester of Year 1. Teaching mostly impacted only spring implementers who started after February 2020. There were very few cases in which this happened. For the most part, most officers in elementary schools where in-school learning shifted to online learning were able to finish teaching lessons on zoom. Overall, of 38 schools participating in the project, 15 had completed teaching before the pandemic, 13 schools completed online teaching, 10 were lost to the project because of COVID. Year 2 started in the fall of 2020. By then, most elementary schools had shifted to online learning and the program was implemented almost universally using online instruction with 31 schools completing the program online.

### Study design and retention

The study design called for students who received the elementary version of *kiR* from D.A.R.E. officers to be pretested prior to implementation of the program. Students were to be posttested immediately upon their completion of the 10-session program; follow-up testing occurred in their subsequent school year. Year 1 and Year 2 data were combined for analysis.

The COVID-19 pandemic occurred during March 2020 and significantly impacted retention at posttest and follow-up later in the spring of 2020. In the end, 2,049 students (33.5%) completed analyzable pretest and posttest surveys and 1,486 (24.3%) completed analyzable pretest and follow-up surveys.

### Measures

The student survey included dichotomous assessments of past 30-day alcohol use, drunkenness, cigarette smoking, and vaping. Students also responded to prompts about their beliefs about the consequences of smoking and drinking (3 items), intentions to avoid alcohol, cigarette, and marijuana use (3 items), normative beliefs about the prevalence and acceptability of alcohol and cigarette use (4 items), and their ease or difficulty related to refusing offers to drink alcohol, smoke cigarettes, and use marijuana (3 items). Responses to each prompt were formatted with values ranging from 0-to-10 with values of 10 being the most theoretically desirable. For example, “strongly agree” responses to the prompt, “I have made a final decision to stay away from marijuana” were coded as 10. Similar responses to, “If I drank alcohol at my age, it would hurt my body” were also coded as 10. The normative belief question, “How many people your age do you think get drunk at least once a month?” was coded 10 if students responded “none.” We averaged students’ responses to these 13 items to form a psychosocial scale (Psych; α = .681). The psychosocial scale also had a range of 0-to-10.

Classroom teachers were tasked with completing fidelity assessment forms for each lesson. They (1) assessed whether objectives had been achieved, (2) the proportion of within-lesson activities that were completed, and rated on 0–10 scales how (3) prepared and (4) energetic the officer was, (5) student attentiveness, (6) student engagement, and (7) the level of classroom control.

### Analysis plan

#### Virtual controls

The fundamental reason for including control groups in prevention research is to define the outcomes that would result had no intervention been delivered. It is increasingly difficult to find suitable control groups for evaluating school-based drug prevention programs. Among the barriers that limit control group recruitment have been an increase in priorities schools are placing on meeting basic educational requirements. COVID-19 has further focused educational priorities. Programs like D.A.R.E. that are widely disseminated further hamper the ability of researchers to randomize to condition and have fully naïve control students to compare to. Even when included, there is no guarantee that control groups will fulfill their fundamental purpose. Selection bias and differential attrition are among the known threats to internal validity.

Our solution to this dilemma is to use the virtual controls methodology [26] as a comparison group. Because this is the first prospective study in which virtual controls is used, some explanation is warranted. A simplified explanation of virtual controls can be viewed at https://vimeo.com/486993156. We developed the virtual controls as a potential remedy to the afore mentioned challenges [28]. Virtual controls is based on two findings from our previous analysis of 344,429 surveys collected from 106,470 research participants pooled from 25 research studies that were normalized, harmonized, and pooled for analysis [29]. The virtual controls’ data are publicly available from 10.5281/zenodo.5256140. First, the prevalence of past 30-day alcohol drinking, drunkenness, cigarette smoking, marijuana use, and the prevalence of many other substances regularly increases with age throughout adolescence. Prevalence rates can range between zero (0) and one (1) with a lower prevalence preferred. Second, there are a set of psychosocial variables that are highly correlated with these behaviors that can be used as proxy measures of substance use. Psychosocial variables were coded so that the range for each varied between zero (0) and ten (10) with high values being theoretically more desirable. This creates a situation in which prevalence and psychosocial scores will be inversely correlated.

Our first step in creating virtual controls was create a single variable (psych score) by selecting and combining the set of psychosocial indices that best predicted substance use behaviors. Intentionality, peer descriptive normative beliefs, peer injunctive norms, beliefs about the positive and negative consequences of use, and attitudes were demonstrated to have the potential to serve as proxies in the assessment of substance use risk [29]. We calculated psych scores based on 284,404 surveys that included one or more of these variables.

**Fig 1:**
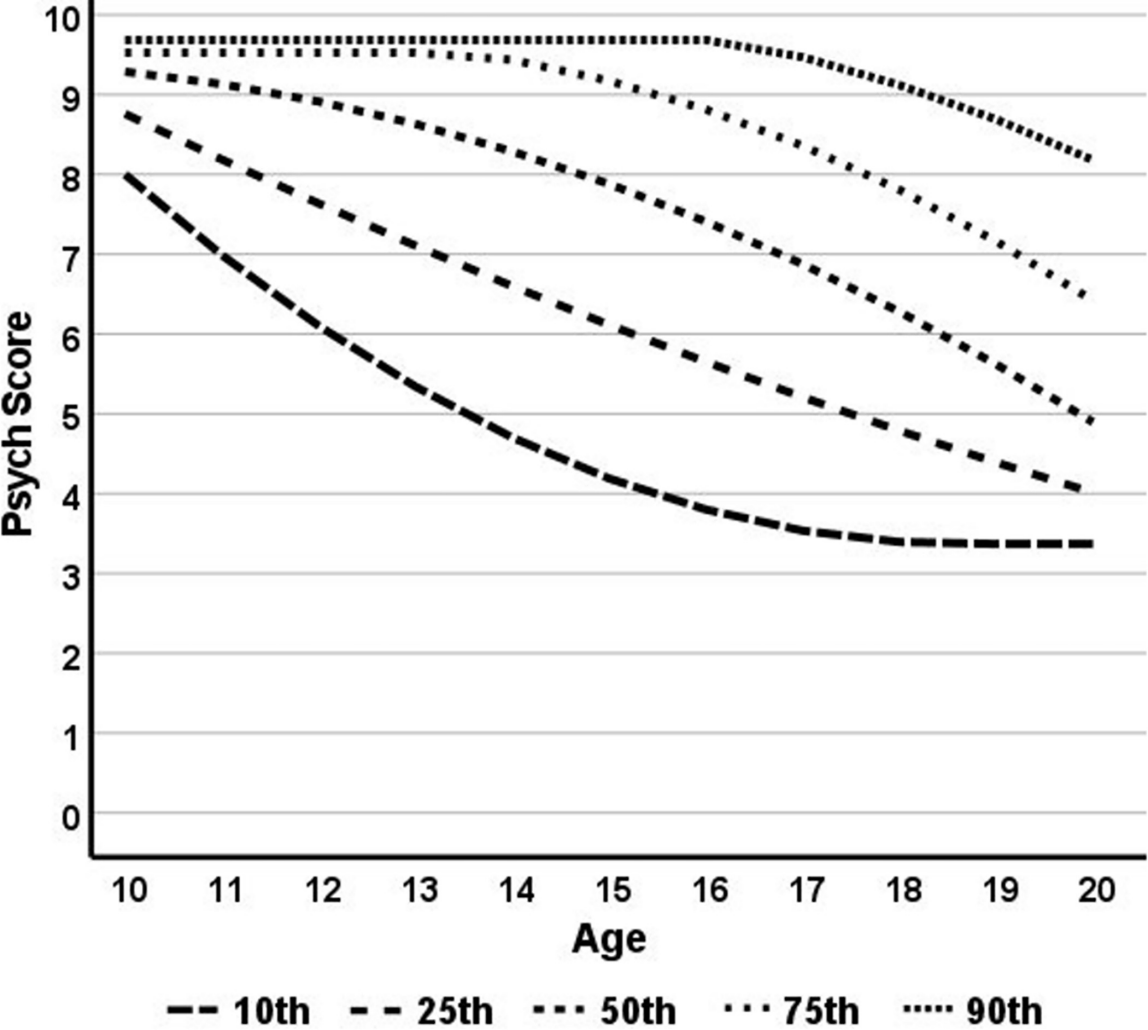
Changes in psych scores across ages for five sample percentiles.

The second step was to model age-related variations in psych scores for each percentile from the 0.5^th^ through the 95.5^th^ percentiles. Age was calculated in months, ranging from 120 months (10 years of age) through 240 months (20 years of age). This resulted in 23,958 data points (198 percentiles x 121 ages). In order to systematize and “smooth” percentiles, quadratic functions were applied to each. Fig 1 presents psych scores associated with a sample of five percentiles (10^th^, 25^th^, 50^th^, 75^th^, and 90^th^). As can be seen, all psych scores decline with declines most rapid among lower percentiles.

The third step involves using data from the live evaluation project. Using whatever psychosocial data are available through the live evaluation, a single psych score is calculated for each participant for each wave of data collection. Ideally, a set of variables similar to the ones included in step two is included in the creation of the psych score and that this reflects an optimized correlation between combined psychosocial measures and assessed behaviors.

The virtual controls algorithm combines data from steps two and three. The virtual controls algorithm [28] relies on logistic regression results associated with the treatment group included in the analysis. B weights are calculated using pretest data from treated students. The formula for calculating probability of use is:

$$Fp(x)=\frac{e^{t}}{1+e^{t}}$$

where *Fp*(*x*) is the expected probability of a substance use behavior and *t* is:

$$B_{Intercept}+B_{Psych}\times Psych+B_{\left(Psych\times Age\right)}\times (Psych\times Age)$$

The estimated probabilities of behavior for each age x percentile entry combined with step two results are incorporated into a table. The algorithm sorts through this table to identify the percentile that most closely matches each treatment case’s pretest age and psych score. A virtual case is then created that is pegged to the percentile of the match. Using the treatment case’s age at posttest and follow-up and the data associated with virtual case’s percentile, the virtual case’s probabilities of behavior are captured from the table. Thus, each virtual control case’s percentile is used to estimate probabilities of use at posttest and follow-up.

As an example of how the algorithm processes cases, consider student X. At pretest she was 127 months old, 129 months old at posttest, and 137 months old at follow-up. The participant’s psych score based on an analysis of her completed pretest survey was 8.97. Her age and pretest psych score were used to find a match in the virtual controls table and pegged the virtual control case to be at the 41st percentile. At posttest and follow-up, the virtual control case remained at the 41st percentile. Psych scores for this percentile were 8.93 at posttest when student X was 129 months old and 8.73 when student X was 137 months old. The virtual control case’s probability of drinking, smoking, and using marijuana were based on applying B weights from the project’s logistic regression outcome to these ages and psych scores. This virtual control case’s probabilities of drinking at pretest, posttest, and follow-up were 1.20%, 1.58%, and 4.61%, respectively. The probabilities of her getting drunk were at pretest, posttest, and follow-up were 0.02%, 0.02%, and 0.03%, respectively. The probabilities of vaping were 0.04%, 0.04%, and 0.05%, respectively.

It is worth noting that the live student’s behavioral self-reports and psych scores were assessed using the survey that was administered to students who received the intervention. Except for the pretest psych score and the student’s ages, her actual behaviors were independent of the estimates of the virtual control case. Therefore, her own behaviors, were based on her self-reports at pretest, posttest, and follow-up and coded as either no (0) or yes (1). Her posttest and follow-up psych scores were also based on the self-reports she provided to administered surveys. Neither her self-reported behaviors nor her posttest and follow-up psych scores were used in the algorithm that created the virtual control case.

Table 1 presents sample percentiles linked to ages (in months) and psych scores. Virtual control cases whose treatment students’ initial psych score would place them in the 25th percentile have subsequent psych scores that decline markedly as they grow older. The virtual control case for a student whose initial psych score was at the 50th percentile would have relatively less decline over time. The virtual control case for a student at the 75th percentile would see no decline until after 163 months.

**Table 1:** Virtual controls’ sample percentiles associated with various ages and psych scores.

Age (Months)	25th Percentile	Difference*	50th Percentile	Difference	75th Percentile	Difference
120	8.7601		9.2878		9.5261	
126	8.4713	-0.2888	9.2182	-0.0696	9.5261	0.0000
132	8.1879	-0.5723	9.1328	-0.1551	9.5261	0.0000
138	7.9099	-0.8502	9.0316	-0.2563	9.5261	0.0000
144	7.6373	-1.1228	8.9146	-0.3732	9.5261	0.0000

* Cumulative difference from age 120 months.

#### Nested analyses: Students within classrooms within schools

Differences between D.A.R.E. and virtual controls were assessed and t-test significance, and Cohen’s *d* were reported for each behavior and each wave of data (pretest, posttest, and follow-up). Analyses were planned to assess the impact of D.A.R.E. instruction at both individual student, classroom, and school levels. Once virtual control cases were created, each was assigned a parallel classroom and school identifier to its corresponding treatment case.

To validate outcomes, in addition to the t-test and Cohen’s *d* analyses, multi-level model (MLM) analyses were performed that took the classroom and school covariance structure into account. Race, gender and ethnicity were included as covariates. Pretest to posttest and pretest to follow-up differences from both virtual control and treatment groups were dichotomized to represent an increase in drug use from pretest to posttest and follow-up.

#### Respondent subgroup analyses

In addition to overall D.A.R.E. versus virtual control analyses, we also completed subgroup analyses separately for boys, girls, Hispanics, Non-Hispanics, Whites, and Non-Whites. To complete these analyses, virtual control cases were assigned the same gender and race/ethnicity as their matched treatment cases. Therefore, for each of these subgroup analyses, the treatment subgroup was compared to the corresponding matched set of virtual control cases.

## Results

### Fidelity

On average classroom teachers judged 94.7% of objectives to have been achieved and 94.5% of activities to have been completed. Teachers rated officers as being prepared (mean = 9.92, st dev = 0.64) and energetic (mean = 9.52, st dev = 1.45). Students were rated as mostly attentive (mean 8.98, st dev = 2.08) but somewhat less engaged (mean = 7.58, st dev = 2.57).

### Virtual controls’ parameters

As noted in Methods, virtual controls uses logistic regression B weights derived from the treatment group’s pretests to estimate age x percentile substance use probabilities. As might be deduced from the use of logistic regression, when there is no use or very low prevalence of use at pretest, the weights in the formula become unstable. In our analyses, there were too few cases of students reporting past 30-day cigarette smoking to complete virtual control estimates. As a result, the only estimates that could be calculated were for alcohol use, drunkenness, and vaping. Table 2 summarizes B weights for these three behaviors.

**Table 2:** B weights used for estimating the probability of use for three substances.

	Alcohol	Drunkenness	Vaping
Psych	-2.421	-0.900	-0.801
Age x Psych	0.014	0.000	0.000
Constant	1.361	2.092	1.684

**Fig 2:**
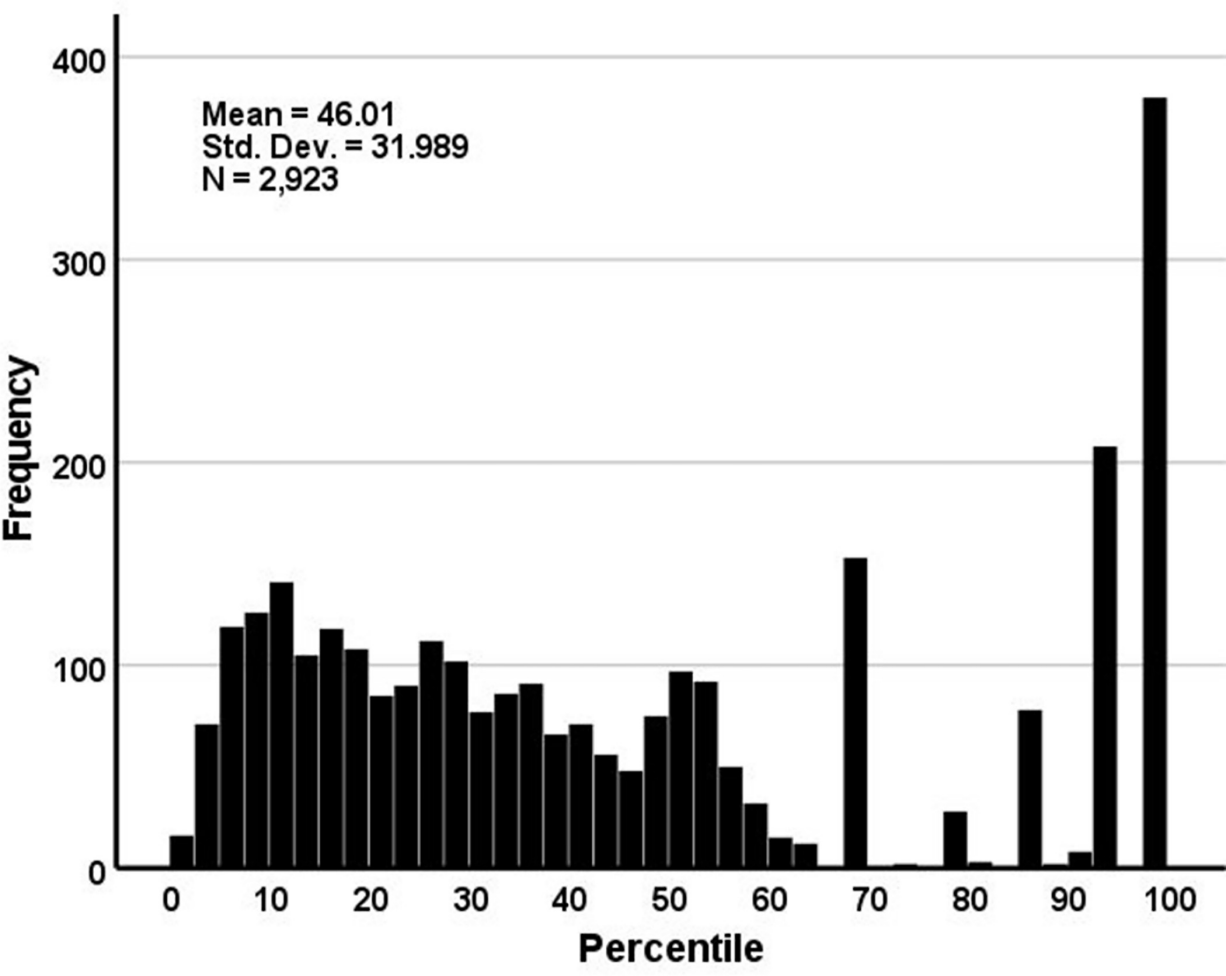
Distribution of virtual control cases’ assigned percentiles.

The algorithm pegged virtual cases’ percentile ranks based on treatment cases’ pretest age and psych score. The resulting distribution of percentile ranks is presented in Fig 2. As can be inferred, there were a sizeable number of cases (13.0%) had the highest possible percentile rankings. However, most (about 70%) had percentile assignments between the 0.5^th^ and the 60.0^th^ percentiles.

### Psychosocial and behavioral outcomes

**Fig 3:**
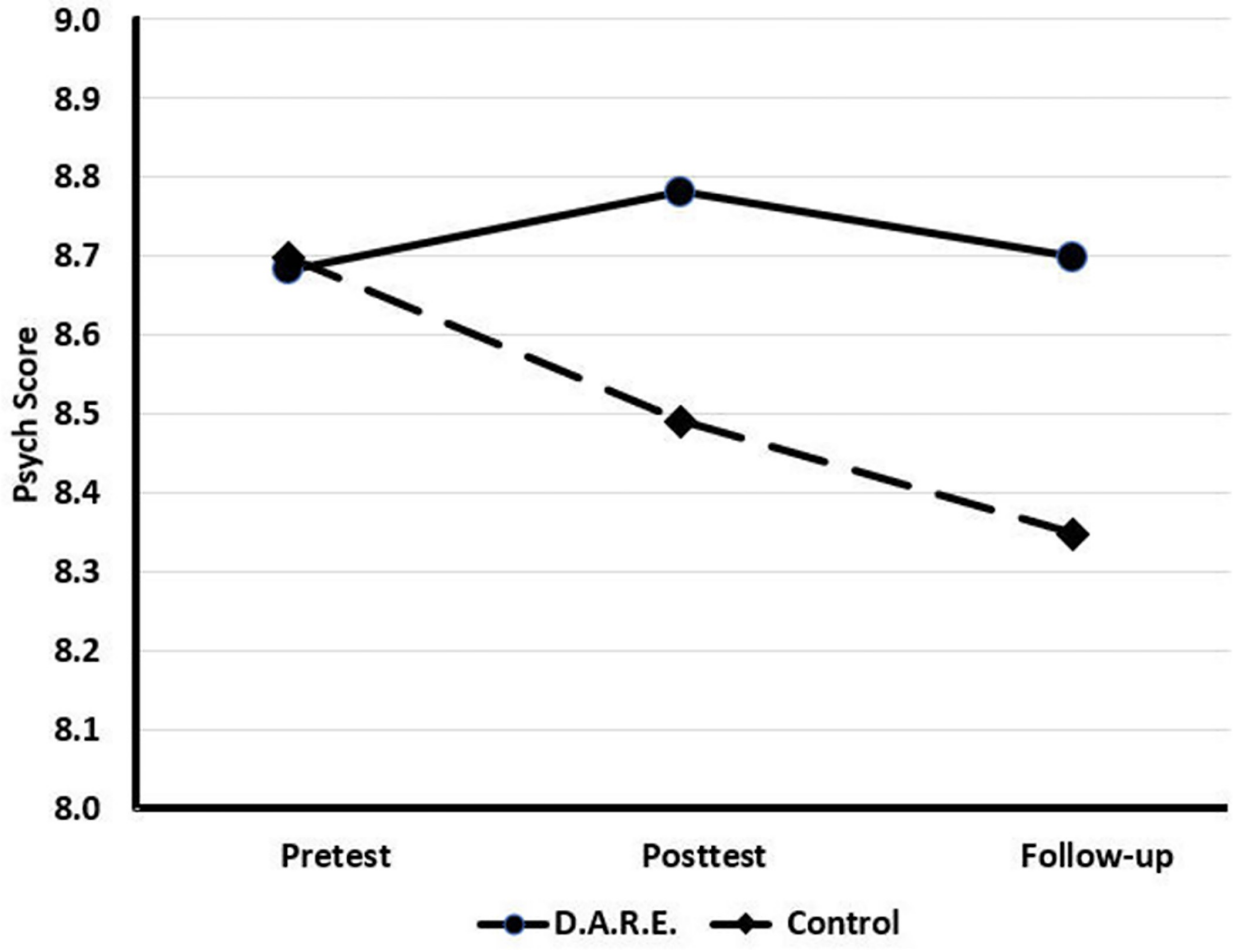
Comparison of psych scores for students receiving D.A.R.E. and virtual control cases.

Psych scores were nearly identical for D.A.R.E. and virtual control cases at pretest (8.68 vs 8.70, respectively, *t* = 0.620, *df* = 5,844, *p* = .535). Thereafter, D.A.R.E. students’ psych scores improved whereas virtual control cases’ scores gradually declined. At posttest, D.A.R.E students’ psych scores were 8.78 compared to control’s psych scores of 8.49 (*t* = -8.187, *df* = 4,085, *p* < .001). Similarly, at follow-up D.A.R.E. students’ and controls’ scores were respectively 8.70 and 8.35 (*t* = -7.682, *df* = 2,944, *p* < .001; see Fig 3).

Table 3 presents a summary of comparisons between D.A.R.E. students’ self-reported prevalence and the virtual controls’ estimates of probabilities of drinking alcohol, getting drunk, and vaping at pretest, posttest, and follow-up. Outcomes are presented at the student level, aggregated at the classroom level, and aggregated at the school level. At the student level, there were statistically significant differences between D.A.R.E. students and virtual control estimates for all three substances; however, based on the interpretation of Cohen’s *d*, differences were trivial. The magnitude of difference grew, but only slightly, at the immediate posttest. For drinking alcohol, the follow-up difference between groups became meaningfully large, with estimated use among virtual control cases rising above 9%. Drunkenness and vaping rose among virtual control cases as well, but not to the same degree.

**Table 3:** D.A.R.E. versus virtual control behavioral outcomes for pretest, posttest, and follow-up.

Student-Level	D.A.R.E.	Control	*t-test*	*df*	*p*	Cohen’s *d*
Alcohol Pretest	1.65%	2.42%	0.032	5,592	0.001	0.086
Alcohol Posttest	1.77%	4.82%	9.649	3,937	0.001	0.308
Alcohol Follow-up	1.77%	9.67%	19.142	2,813	0.001	0.722
Drunkenness Pretest	0.60%	0.57%	-0.191	5,597	0.849	-0.005
Drunkenness Posttest	0.68%	0.81%	0.686	3,934	0.492	-0.041
Drunkenness Follow-up	0.44%	1.08%	3.448	2,811	0.001	0.130
Vaping Pretest	0.82%	0.78%	-0.235	5,840	0.814	-0.006
Vaping Posttest	1.02%	1.05%	0.120	4,078	0.904	0.004
Vaping Follow-up	0.67%	1.34%	2.993	2,942	0.003	0.110
Class-Level						
Alcohol Pretest	1.54%	2.46%	3.352	444	0.001	0.318
Alcohol Posttest	1.64%	4.69%	8.070	329	0.000	0.888
Alcohol Follow-up	1.83%	10.66%	14.170	269	0.000	1.723
Drunkenness Pretest	0.54%	0.58%	0.273	444	0.785	0.026
Drunkenness Posttest	0.54%	0.77%	1.402	329	0.162	0.154
Drunkenness Follow-up	0.46%	1.20%	3.363	269	0.001	0.409
Vaping Pretest	0.82%	0.80%	-0.138	464	0.890	-0.013
Vaping Posttest	0.71%	1.01%	1.685	342	0.093	-0.030
Vaping Follow-up	0.57%	1.48%	3.825	279	0.001	0.456
School-Level						
Alcohol Pretest	1.50%	2.46%	2.891	80	0.005	0.639
Alcohol Posttest	1.73%	4.84%	6.217	73	0.000	1.436
Alcohol Follow-up	1.85%	9.87%	10.194	60	0.000	2.589
Drunkenness Pretest	0.85%	0.58%	-0.881	80	0.381	-0.195
Drunkenness Posttest	0.82%	0.83%	0.023	73	0.982	0.005
Drunkenness Follow-up	0.39%	1.14%	3.970	60	0.001	1.008
Vaping Pretest	0.79%	0.78%	-0.012	80	0.990	-0.003
Vaping Posttest	0.94%	1.07%	0.481	74	0.632	0.110
Vaping Follow-up	0.52%	1.40%	3.541	60	0.001	0.899

A similar picture emerged for class-level findings. While drinking alcohol was different between conditions at pretest, the differences increased markedly at posttest and follow-up. For drunkenness and vaping at the classroom level, there were no significant differences at pretest, increasing differences at posttest, and relatively greater differences at follow-up.

To a great extent, school-level findings mirror the classroom-level findings. Drinking alcohol differed between D.A.R.E. and virtual control aggregated cases at pretest with differences between the two groups increasing at posttest and follow-up. Drunkenness and vaping mirrored classroom-level results with non-significant differences at pretest and posttest and significant differences at the follow-up.

Of the 36 subgroup analyses (six subgroups, three outcomes, and two test periods), 13 yielded significant z-test outcomes at the 95% confidence level. The remainder were not significant and therefore essentially the same for D.A.R.E. students and associated virtual control cases. These are summarized in Table 4. In all cases, D.A.R.E. students had less prevalence than did estimates for virtual controls. It should be noted that the Non-Hispanic category included Black, White, Asian, Pacific Island, Native American, and “Other”. While there were no differences between Hispanic students and their virtual control cases, for all other students there were differences for all other groups when combined into one ethnic group category when compared to these students’ virtual control cases.

**Table 4:** Subgroup analyses where D.A.R.E.-exposed student differed significantly from virtual controls*.

Subgroup	Behavior	Test	D.A.R.E.	Controls	p-value
Boys	Alcohol	Follow-up	0.8%	4.0%	0.000
Boys	Vaping	Follow-up	0.6%	2.2%	0.015
Girls	Alcohol	Posttest	0.1%	1.1%	0.009
Girls	Alcohol	Follow-up	1.3%	3.0%	0.036
Girls	Vaping	Follow-up	0.3%	1.4%	0.034
Non-Hispanics	Alcohol	Posttest	0.1%	1.1%	0.000
Non-Hispanics	Alcohol	Follow-up	0.6%	3.4%	0.000
Non-Hispanics	Drunkenness	Follow-up	0.0%	0.7%	0.008
Non-Hispanics	Vaping	Follow-up	-0.1%	1.7%	0.000
Non-Whites	Alcohol	Follow-up	-0.6%	4.0%	0.000
Whites	Alcohol	Posttest	0.2%	1.0%	0.055
Whites	Alcohol	Follow-up	0.8%	3.1%	0.001
Whites	Vaping	Follow-up	0.2%	1.4%	0.008

* Tested at the 95% confidence limit. Non-significant comparisons are excluded.

Table 5 shows the results of the multi-level model analyses taking the classroom-school correlation structure found in the data into account. Looking at the fixed effects shows that the virtual control group had higher levels of drug use compared to the treatment group for alcohol, drunkenness, and vaping for both posttest differences and follow-up differences. Race was significant for drunkenness and vaping at posttest and follow-up with White participants having lower increase in drug use compared to non-White participants. Ethnicity was not found to be a significant factor in any of the analyses. Girls had lower increase in drug use compared to boys for vaping at posttest at follow-up with an effect seen with drunkenness only at posttest.

**Table 5:** Multi-level models: Regression fixed effects.

Behavior	Test	Effect	Estimate	SE
Alcohol	Posttest	D.A.R.E. vs Control	12.25	0.63
Drunkenness	Posttest	D.A.R.E. vs Control	4.61	0.35
Drunkenness	Posttest	White	-0.38	0.13
Drunkenness	Posttest	Girls vs Boys	-0.27	0.12
Vaping	Posttest	D.A.R.E. vs Control	5.32	0.26
Vaping	Posttest	White	-0.29	0.12
Vaping	Posttest	Girls vs Boys	-0.25	0.09
Alcohol	Follow-up	D.A.R.E. vs Control	11.37	0.91
Drunkenness	Follow-up	D.A.R.E. vs Control	4.75	0.47
Drunkenness	Follow-up	White	-0.47	0.16
Vaping	Follow-up	D.A.R.E. vs Control	5.62	0.37
Vaping	Follow-up	White	-0.35	0.13
Vaping	Follow-up	Girls vs Boys	-0.37	0.11

* Tested at the 95% confidence level. All models took classroom and school covariance into account.

## Discussion

This paper summarizes the evaluation of D.A.R.E. officers’ delivery of the elementary version of *kiR*. Results indicate that when compared to an algorithmically generated control condition, the program had identifiable improvements in both a key psychosocial measure and in terms of deterring the onset of 30-day alcohol use, drunkenness, and vaping. These outcomes were observed at the immediate posttest and a one-year follow-up test. They provide evidence that the delivery of elementary school *kiR* by D.A.R.E. officers is having a positive effect. The program was effective at some level for girls and boys, for non-Hispanics, for non-Whites (in one case–alcohol use at follow-up) and for Whites.

As substance use prior to the age of 15 is associated with a number of negative consequences (i.e. substance abuse and dependence, changes in brain functioning, poor cognitive performance, high rates of mood disorders), prevention science often directs interventions to reduce use or delay the onset of substance use for adolescents [30]. As was expected, there was very low prevalence of cigarette use, alcohol use, drunkenness, and vaping among the students. Compared to virtual controls, at multiple time points over the course of a calendar year, elementary students who received D.A.R.E. lessons decreased use and continued to not use alcohol and other substances.

We wish to point out a number of limitations of this study. The prevalence of substance use among students was extremely low. Fifth grade students in this sample essentially did not have experience with recent (30-day) use at pretest, posttest, or follow-up. Such low rates of use are gratifying from a public health perspective. Nonetheless, prevention programs are most meaningfully evaluated when the prevalence of use is otherwise expected to increase beyond what was observed. An evaluation that followed students into later years would perhaps resolve this.

This study used a virtual controls algorithm to estimate what the normal development of substance use would be like. In an environment where experimental controls may be difficult to recruit and retain, this offered a reasonable alternative to the traditional approach to evaluation [31]. Even when quasi-experimental and randomized field trials are conducted, there are often threats to internal validity based on selection and retention. For example, it is not unusual for there to be pretest non-equivalence when comparing treatment and control conditions. In this study, treated students were matched precisely with virtual cases, eliminating selection bias of the comparison group as a threat to internal validity. For each treatment student at each wave of data collection, an age-matched virtual control case was generated. Differential attrition is also common among control groups, with control groups often having greater attrition than treatment groups [32, 33]. Using the virtual controls methodology, there was no differential attrition between treated students and their virtual cases; all treated cases had virtual control matches when they were surveyed at posttest and follow-up. Moreover, the changes observed over time in the virtual control group appeared to be reasonable in terms of changes in the psych scores as well as estimates of prevalence.

Nonetheless, there was extreme attrition in the sample available for analysis. This was primarily attributable to schools closing during the COVID-19 pandemic. The primary source of attrition was schools not being able to participate rather than students declining to participate in subsequent survey administrations. Despite the degree of attrition, a comparison of all students who completed pretests with those who were included only in pretest-posttest and pretest-follow-up analyses were remarkably similar. Psych scores were in the same general range. Prevalence of use was slightly lower for students included in analyses. However, because prevalence rates were so low, in any other context there would be no practical difference. Nonetheless, students included in pretest-follow-up analyses were slightly more conventional than either the entire pretest sample or the pretest-posttest sample.

When the virtual controls method was designed, age limits needed to be placed on the percentile database because none of the studies included participants who were younger than 120 months. Students younger than 120 months old were therefore excluded from analysis. When prevalence is near zero, the reliability of logistic regression weights diminishes. We noted that the pretest correlation between psych scores and self-reports of use were generally weaker than we have otherwise experienced [29]. For instance, in the dataset that pooled 25 studies, the correlation between 30-day alcohol use and psych score was -.524. The same correlation in this study’s pretest sample was -.149. Stronger relations between psych scores and behaviors are expected to produce more reliable outcomes.

It should be noted that there were typical issues related to completeness of the data. Student data collection was completed at remote sites under the direction of school personnel. In cleaning the data, there was some researcher-induced attrition due to such issues as duplicate ID numbers, lack of consistency with birth dates and genders across waves of data, and a variety of missing data issues. These were not beyond what normally occurs in field research of this type but should nonetheless be noted.
